# Self-Powered Humidity Sensor Driven by Triboelectric Nanogenerator Composed of Bio-Wasted Peanut Skin Powder

**DOI:** 10.3390/polym16060790

**Published:** 2024-03-13

**Authors:** Muhammad Saqib, Shenawar Ali Khan, Maryam Khan, Shahzad Iqbal, Muhammad Muqeet Rehman, Woo Young Kim

**Affiliations:** Department of Electronic Engineering, Jeju National University, Jeju 63243, Republic of Korea; saqibmuhammad@jejunu.ac.kr (M.S.); shenawaralikhan@jejunu.ac.kr (S.A.K.); maryamkhan93@stu.jejunu.ac.kr (M.K.); shahzadiqbal@stu.jejunu.ac.kr (S.I.)

**Keywords:** self-powered sensor, peanut skin bio-waste, triboelectric nanogenerator, versatile humidity sensor

## Abstract

The increasing number of IoT devices has led to more electronic waste production, which harms the environment and human health. Self-powered sensor systems are a solution, but they often use toxic materials. We propose using biocompatible peanut skin as the active material for a self-powered humidity sensor (PSP-SPHS) through integration with a peanut-skin-based triboelectric nanogenerator (PSP-TENG). The PSP-TENG was characterized electrically and showed promising results, including an open circuit voltage (162 V), short circuit current (0.2 µA), and instantaneous power (2.2 mW) at a loading resistance of 20 MΩ. Peanut skin is a great choice for the sensor due to its porous surface, large surface area, eco-friendliness, and affordability. PSP-TENG was further used as a power source for the PSP-humidity sensor. PSP-SPHS worked as a humidity-dependent resistor, whose resistance decreased with increasing relative humidity (%RH), which further resulted in decreasing voltage across the humidity sensor. This proposed PSP-SPHS exhibited a good sensitivity (0.8 V/RH%), fast response/recovery time (4/10 s), along with excellent stability and repeatability, making it a potential candidate for self-powered humidity sensor technology.

## 1. Introduction

Humidity sensors are widely used in several industries, including agriculture, pharmaceuticals, cosmetics, textiles, food, and beverage. They play a significant role in continuous environmental monitoring in the Internet of Things (IoT) industry and smart homes [1,2,3,4,5,6]. Furthermore, humidity sensors integrated into e-skin systems contribute significantly to expanding the capabilities of wearable monitoring devices by enhancing sensory perception, improving monitoring accuracy, and ensuring biocompatibility and transparency for diverse applications in healthcare, military, and environmental sectors [7,8]. Typically, humidity sensors rely on replaceable batteries for power, which can be costly and inconvenient to replace. This leads to the accumulation of electronic waste (E-waste), posing environmental burdens. Improper disposal of E-waste results in ecosystem contamination, landfill space depletion, resource wastage, increased energy consumption, and global environmental impacts [9,10,11]. Researchers have begun to develop self-powered humidity sensors to cut down the need for external power supplies [12,13]. Presently, several self-powered humidity sensors (SPHSs) exist, including piezoelectric nanogenerators [14], triboelectric nanogenerators [15], humidity-induced voltage sources [13], solar cell integrated humidity sensors [16], and potentiometric humidity-transduction [12]-mechanism-based SPHSs.

Among these different types of SPHSs, triboelectric nanogenerator (TENG)-based SPHSs received more attention due to their simple device structure, versatility, high sensitivity, scalability, easy fabrication, high performance, and economic feasibility [17,18,19,20]. Recently, many researchers reported TENG-SPHS, using different types of materials like PTFE [21], chitosan [22], reduced graphene oxide (RGO)-polyvinylpyrrolidone (PVP) [18], tin disulfide nanoflowers/RGO [15], chitosan/amido-graphene oxide [23], titanium oxide nanotube arrays [24], graphene oxide (GO) paper [25], GO nanoribbons [26], fluorinated ethylene propylene (FEP)/nylon-66 (PA66) [19], chitosan/activated carbon [27], and perfluorosulfonic acid ionomer (PFSA)/fluorinated ethylene propylene (FEP) [25]. The main objective of promoting SPHSs is to replace conventional sensors that require a constant external power source like replaceable batteries, which leads to E-waste and harms the environment. Alternatively, SPHSs can produce their own power from ambient energy (humidity, mechanical, thermal, etc.) to fulfill their requirements. While the demonstrated SPHSs function effectively, the materials utilized often lack biocompatibility, which remains a significant unresolved concern for us [12,16,22,24,25] due to which biodegradable materials need to be studied for TENG [28,29] and humidity sensors [30,31,32,33]. Cellulose and lignin, which are biocompatible materials, demonstrate impressive performance in TENGs [34,35,36,37] and also exhibit good humidity-sensing properties [36,37,38]. With the motivation to find a solution, we explored the properties of peanut skin, a bio-waste rich in cellulose (46%) [38] and lignin (23%) [39], for use in a SPHS.

This study presents a successful demonstration of a biocompatible SPHS integrated with a TENG device by utilizing peanut skin powder as the primary material. The TENG, comprising a biocompatible PSP (tribopositive layer) and PTFE (tribonegative layer), effectively generated a high voltage (162 V) and power (2.2 mW). The resistive humidity sensor based on PSP exhibited notable characteristics, including good sensitivity (0.8 V/%RH), fast response/recovery times (4/10 s), high stability, and good repeatability. These findings established PSP as a promising material for self-powered humidity sensor technology.

## 2. Experimental Section

### 2.1. Materials

Peanuts were purchased from the open market of Jeju Island, South Korea. The peanuts were carefully removed and ground to obtain peanut skin powder (PSP). Aluminum (Al) tape, copper (Cu) tape (40 µm thickness), polytetrafluoroethylene (PTFE) sheet (30 µm thickness), and polyethylene terephthalate (PET) (100 µm thickness) were purchased from 4Science Co. Ltd., Seoul, Republic of Korea. All the materials were used without additional processing.

### 2.2. Device Fabrication

**Figure 1:**
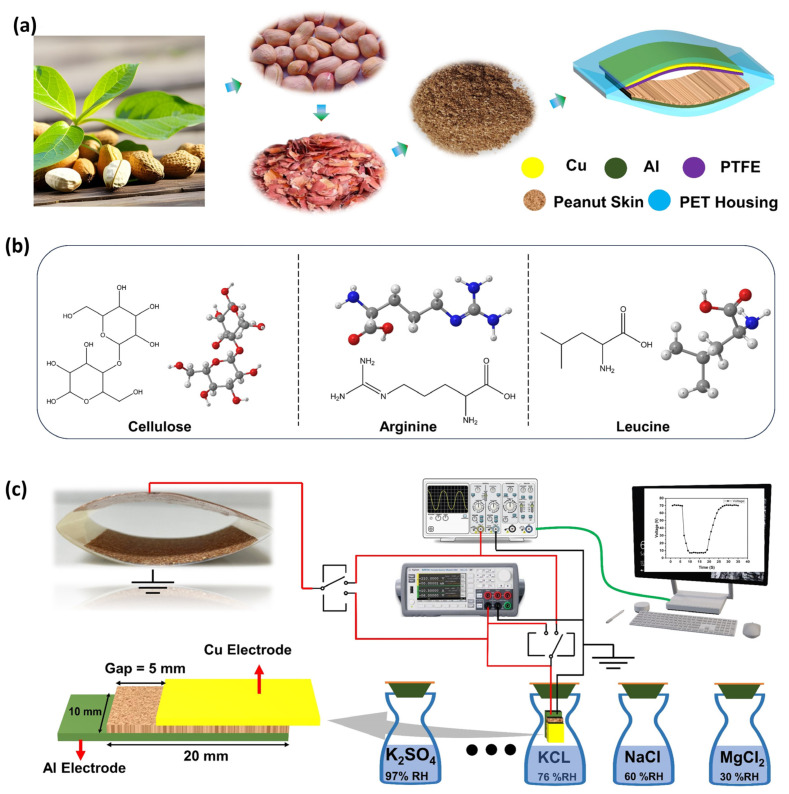
Setup schematic; (**a**) schematic of PSP-TENG; (**b**) chemical structure of different elements that exist in peanut skin; (**c**) electrical characterization setup used for PSP self-powered humidity sensor.

We fabricated two different devices (a resistive humidity sensor and a TENG) separately, which were then integrated with each other to form a SPHS. The complete process of achieving PSP is shown in Figure 1a. It was observed that the fabricated TENG device worked in the contact separation mode by using PSP (tribopositive) and PTFE (tribonegative) thin films as a triboelectric pair. Aluminum (Al) and copper (Cu) tape were used as highly conductive electrode materials, while PET sheet was used as a supporting layer for both sides of PSP-TENG, as shown in Figure 1a. For one side of the TENG, Al tape was affixed to a PET sheet using double-sided tape. A uniformly thin layer of the prepared PSP was then deposited onto the adhesive side of the aluminum tape. The second side of the TENG was formed by adhering Cu tape to a PET layer using double-sided tape. A PTFE thin film was then applied to the adhesive side of the copper tape. Both sides of the TENG were then curved and attached together, as shown in Figure 1a. Peanut skin contains various elements, each with its unique atomic structure. These elements mainly include cellulose, arginine, and leucine, whose chemical structures are shown in Figure 1b. The chemical structure of the peanut skin, determined by the arrangement and bonding of its atoms, influences its composition, properties, nutritional content, flavor, and potential applications in diverse fields. The PSP-based humidity sensor had a sandwiched structure, whose schematic diagram and optical image are shown in Figure 1c.

The fabrication process began by attaching aluminum tape as the bottom electrode to a PET substrate using double-sided tape, and then a thin film of peanut skin powder (PSP) was deposited onto the adhesive side of the aluminum tape. As the final step, the top electrode (copper tape) was affixed to the PSP layer, as illustrated in Figure 1c. Both these devices, the PSP-TENG and PSP resistive humidity sensors, were successfully integrated together to form a single unit of PSP-SHS, as shown in Figure 1c. The PSP-TENG output was connected as an input voltage to the PSP humidity sensor. Then, the PSP humidity sensor was placed in different relative humidity %RH environments and its electrical characterization was performed; Figure 1c depicts the electrical measurement setup used to characterize the self-powered humidity sensor. Different salt solutions provide different humidity levels on their surfaces in a closed jar in an ambient environment. As the humidity-dependent sensor was placed in different salt solutions, its resistance changed, which corresponded to the output voltage change that was measured by an oscilloscope (DSOX3014T); more details of this setup can be found in our previous work [40].

### 2.3. Device Characterization and Measurements

The morphology of PSP thin films, its surface roughness, and elemental analysis were investigated by using a field emission scanning electron microscope (FESEM) (TESCAN, Brno, Czech Republic, MIRA3). Fourier transform infrared spectroscopy (FTIR) characterization of the samples was performed by using an instrument from Ettlingen, Germany (BRUKER TENSOR 27) for qualitative and quantitative analysis of PSP, providing information about its chemical composition, molecular structure, and potential functional properties. FTIR spectra were acquired within the spectral range of 500 to 4000 cm^−1^ while employing KBr precipitation conditions.

A linear motor was used to control the force, frequency, and distance of the cyclic contact. This facilitated the control of the applied force, frequency, and distance of travel. The output voltage and current of the TENG device were analyzed using an oscilloscope Keysight DSOX3014T (Santa Rosa, CA, USA), and a precision source measurement unit Keysight 2911A (Santa Rosa, CA, USA), respectively. The electrical characteristics of the PSP-based humidity sensor were assessed by placing it in specific humidity environments created by super-saturated solutions of different salts.

## 3. Result and Discussion

**Figure 2:**
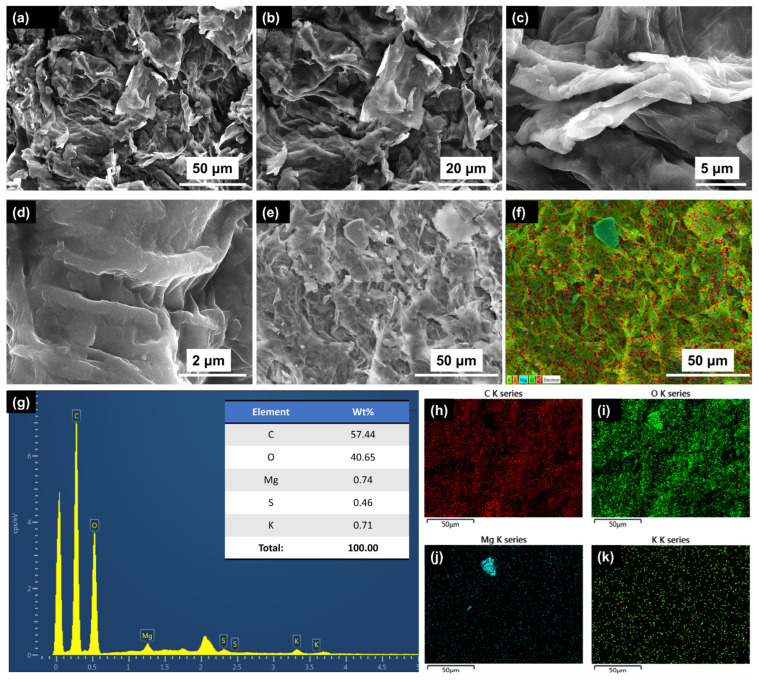
PSP film characterizations. (**a**–**e**) Microstructural analysis of peanut skin powder thin films through FESEM images taken at different resolutions. (**f**) EDS mapping showing the spatial distribution of the elements in PSP thin films. (**g**) EDS analysis of PSP thin film showing quantitatively the wt% of all elements present in it. (**h**) C, K series; (**i**) O, K series; (**j**) Mg, K series; (**k**) K, K series.

Figure 2a–e exhibit the SEM images of the PSP film captured at various resolutions, enabling a detailed examination of its microstructure. An analysis of these images revealed the presence of a uniform PSP film characterized by an extensive surface area, pronounced roughness, a multitude of pores/voids with varying dimensions (which significantly contribute to the film’s permeability and absorption capacity), and irregular pathways for charge carrier conduction. These features of PSP proved it to be a highly suitable material for the targeted applications of TENGs and humidity sensors. In the context of TENGs, the described features of a PSP thin film have significant importance due to their optimized charge transfer, promotion of efficient energy conversion, increase in charge separation, and enhancement in power generation capabilities. These characteristics contribute to the overall effectiveness and performance of the nanogenerator in harnessing mechanical energy and converting it into usable electrical energy. This is supported by research on the use of natural materials, such as sunflower husks [41] and rice paper [42], in the development of high-performance, biocompatible TENGs for biomechanical energy harvesting.

In the context of resistive humidity sensors, the described features of PSP thin films also have a significant impact on their properties. The large surface area of the PSP sensing thin film played a crucial role in enhancing its sensitivity to humidity. This is due to the increased availability of active sites for water vapor adsorption, allowing for improved detection and response to moisture variations. Additionally, the rough surface of the sensing thin film further amplified its effective surface area, promoted greater contact area with the surrounding environment, and enhanced its ability to adsorb water molecules. As a result, the sensitivity and responsiveness to humidity changes were significantly improved. The presence of pores/voids within the sensing thin film also contributed to its performance. These structures enhanced the moisture absorption capacity of the PSP thin film and enabled it to effectively respond to humidity changes. Furthermore, the sensitivity to different humidity levels was enhanced as the pores/voids provided additional sites for water vapor adsorption. The irregular paths for charge conduction within the sensing thin film had a significant impact on its electrical conductivity and response to humidity. These paths influenced the flow of charge carriers, thus affecting the film’s electrical resistance. By measuring the changes in electrical resistance, the sensing thin film can accurately determine the humidity level. Overall, the combination of a large surface area, roughness, pores/voids, and irregular charge conduction pathways contributed to the film’s enhanced sensitivity, responsiveness, and ability to measure humidity levels. The distinctive microstructure of the PSP thin film, as evident from its FESEM images, established its suitability as both a tribopositive layer of a TENG device and a sensing thin film for resistive humidity sensors. The film’s microstructural features, including a large surface area, roughness, numerous pores/voids, and irregular charge conduction pathways, collectively contributed to its functional characteristics and enabled its effective utilization in these applications.

The energy-dispersive X-ray spectroscopy (EDS) analysis of the PSP thin film was performed to analyze its elemental composition. EDS analysis revealed the presence of carbon (C) at 57.44%, oxygen (O) at 40.65%, magnesium (Mg) at 0.74%, sulfur (S) at 0.46%, and potassium (K) at 0.71%, as shown in the obtained EDS graph in Figure 2g. The elemental composition obtained from the EDS analysis was consistent with the expected composition of cellulose [43], hemicellulose [44], and lignin [45], as they are primarily composed of carbon, oxygen, and other trace elements such as Mg, S, and K. Therefore, the EDS analysis results supported the likelihood of the presence of cellulose and lignin in PSP, which are important for its structural and functional properties. The inclusion of cellulose, hemicellulose, and lignin within the sensing thin film of a humidity sensor significantly enhances its moisture adsorption capacity, stability, and durability, rendering it suitable for deployment in sensitive environments and biocompatible applications. The presence of carbon within the film enables efficient charge conduction, which is a critical factor in the resistive humidity sensing mechanism, while the presence of oxygen influences the film’s electrical properties and sensing capabilities. In the context of a triboelectric nanogenerator (TENG), the presence of cellulose, hemicellulose, and lignin within the tribopositive layer contributes to improved triboelectric performance, mechanical integrity, and compatibility with environmentally friendly and biocompatible applications. Additionally, the presence of C in the tribopositive layer enhances electrical conductivity, facilitating efficient charge transport and enhancing the output performance of the nanogenerator. Figure 2f presents the energy-dispersive X-ray spectroscopy (EDS) mapping of the peanut skin powder (PSP) film. The EDS analysis of PSP confirms the presence of the C K series, O K series, Mg K series, and K K series at a magnification level of 50 µm, as illustrated in Figure 2h–k.

**Figure 3:**
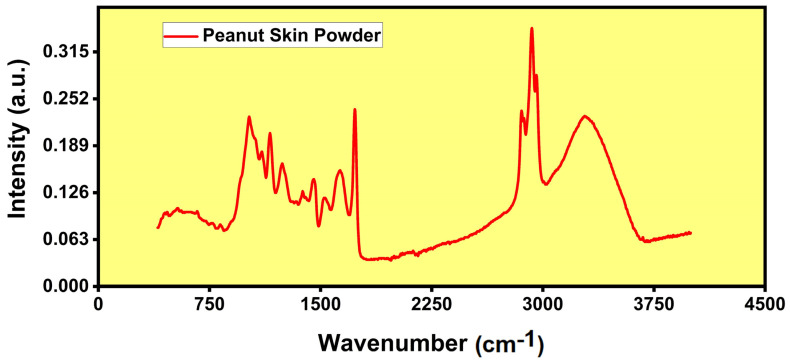
FTIR analysis of peanut skin powder (PSP) thin film.

Figure 3 illustrates the FTIR analysis of PSP thin films. The peaks obtained from the FTIR analysis of peanut skin at different wavemubers of 1000, 1200, 1680, 2800, 2900, and 3300 cm^−1^ were indicative of the presence of cellulose, hemicellulose, and lignin in the skin. The specific peaks corresponded to the characteristic vibrational modes of these components. The peak at 1000–1200 cm^−1^ was associated with the stretching vibrations of C-O and C-C in cellulose and hemicellulose. These natural plant-based elements were suitable for moisture adsorption in a humidity-sensing thin film. The peak at 1680 cm^−1^ was attributed to the stretching vibration of C=O in lignin. The presence of this peak indicated the structural stability and durability of the thin film, essential for long-term humidity sensor performance. The peaks at 2800–2900 cm^−1^ corresponded to the stretching vibrations of C-H in cellulose, hemicellulose, and lignin. These observed peaks are proof of such organic compounds in PSP thin films that were helpful in enhancing the sensing film’s electrical conductivity for the conduction of charge carriers during resistive humidity sensing and TENG. The peak at 3300 cm^−1^ was related to the O-H stretching vibration in cellulose and hemicellulose [46,47]. The observed peak signified the enhancement of the film’s sensitivity to changes in the humidity levels.

**Figure 4:**
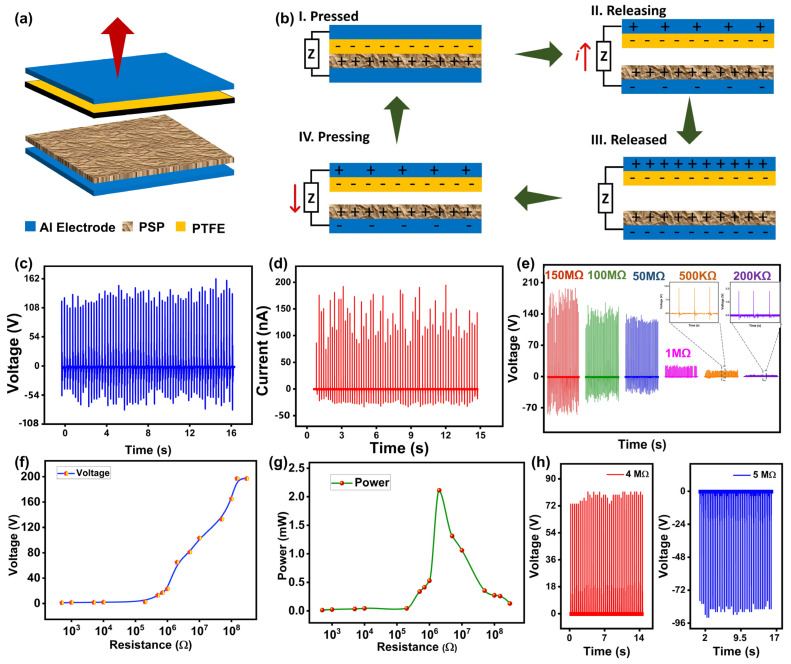
Electrical characterization of PSP-TENG: (**a**) schematic of contact separation mode PSP-TENG; (**b**) working mechanism of CS mode PSP-TENG; (**c**) Voc of PSP-TENG; (**d**) Isc of PSP-TENG; (**e**) voltage waveforms at different loading resistances; (**f**) voltage vs. loading resistances; (**g**) instantaneous power at different loading resistances; (**h**) positive and negative rectified output waveforms.

The schematic of the electrical characterization setup is shown in Figure 1c. The output voltage and current of the PSP-TENG were measured by an oscilloscope Keysight (DSOX3014T) and a precision source measurement unit (KEYSIGHT 2911A), respectively. The fabricated device was a contact separation mode triboelectric nanogenerator (TENG) with an effective contact area of 25 cm^2^. The schematic diagram and conduction mechanism of the TENG are depicted in Figure 4a and Figure 4b, respectively, where the conduction mechanism is divided into four steps. The schematic diagrams illustrate the different stages of the PSP-TENG operation. In the initial neutral state (first stage), no charge was present on the surfaces. When compressed, positive and negative charges were generated and distributed on the PTFE and peanut skin powder films based on their triboelectric tendency (second stage). Upon release, the separated charges formed a dipole moment, driving the flow of electrons from the bottom Al electrode to the top Cu electrode (third stage). This electron flow continued until the maximum separation between the tribopositive and tribonegative sides was reached. When pressed again, the dipole moment vanished, resulting in a reverse flow of electrons between the electrodes (fourth stage). This electrostatic induction process generated output signals until both surfaces were fully in contact again. The output performances of the PSP-TENG were comprehensively investigated.

An energy harvesting assessment was conducted utilizing a linear motor to replicate the cyclic contact separation between two opposing triboelectric surfaces by modifying operational factors like frequency and distance. The voltage output of the TENG was directly gauged using an oscilloscope (DSOX3014T) with an internal load resistance of 10 MΩ. Additionally, the output current was assessed by employing a source measurement unit (Keysight B2911A). The device achieved a peak open circuit voltage of 160 V at a working frequency of 4 Hz and a separation distance of 1 cm at room temperature, as depicted in Figure 4c. Additionally, it generated a short circuit current of 0.2 A, as shown in Figure 4d. The relationship between the output voltage of the PSP-TENG and the loading resistance was explored by measuring the output voltage of the PSP-TENG across six different loading resistances, spanning from 100 Ω to 150 MΩ. Figure 4e shows the output voltage waveforms of the developed device at different loading resistances with the conclusion that the output voltage of the PSP-TENG increased as the loading resistance increased. Figure 4f shows the dependence of the output voltage of the PSP-TENG on loading resistance. The instantaneous power of the device was calculated by the given Equation (1).

(1) $$P=\frac{V^{2}}{R}$$

Figure 4g shows the instantaneous power of the PSP-TENG at different loading resistances, with the conclusion that a maximum power of 2.2 mW at a loading resistance of 20 MΩ could be generated. Figure 4h displays the rectified output voltage waveforms of the PSP-TENG at loading resistances of 4 MΩ and 5 MΩ, showing the positive and negative voltage profiles, respectively.

**Figure 5:**
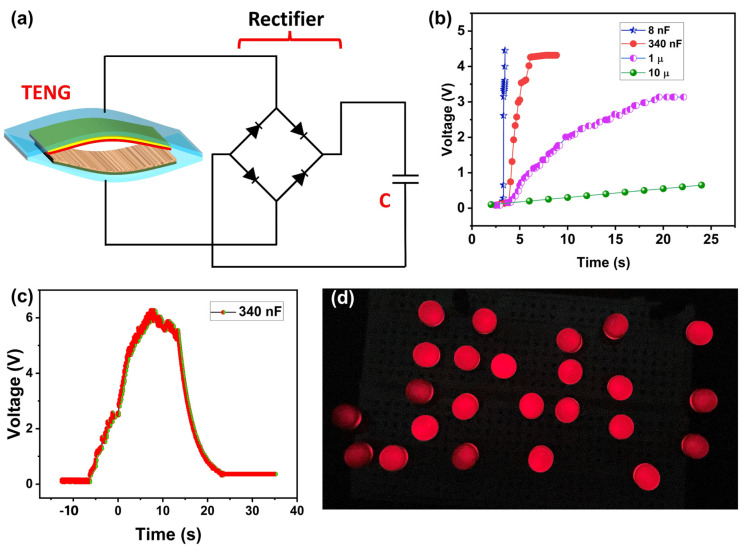
Application of PSP-TENG: (**a**) rectifier circuit to charge capacitors; (**b**) different capacitors charging waveforms; (**c**) charging and discharging of 340 nF capacitor; (**d**) lighting up 24 LEDs by PSP-TENG.

A full-wave rectifier circuit was used to convert the obtained AC waveform of the PSP-TENG into DC voltage for using the developed device in practical applications like charging capacitors and powering different loads, as shown in Figure 5a. Four different capacitors of 10 µF, 1 µF, 340 nF, and 8 nF capacitance, respectively, were charged by using the developed PSP-TENG. Figure 5b shows the charging of these four capacitors. The obtained results showed that the 10 µF capacitor took 20 s to charge up to 1 V, the 1 µF capacitor took 15 s to charge up to 3 V, the 340 nF capacitor took 10 s to charge up to 4.3 V, and the 8 nF capacitor took only 2 s to charge up to 4.5 V. Figure 5c shows the charging/discharging waveform of the 340 nF capacitor. The developed PSP-TENG was capable of charging a 340 nF capacitor to 6 V within a duration of 12 s. Additionally, the PSP-TENG was tested for powering 24 red LEDs, successfully illuminating them using its rectified output voltage. Figure 5d visually depicts the 24 LEDs powered by the PSP-TENG. Supplementary Material S1 provides a video demonstration of the LEDs being powered by the PSP-TENG.

**Figure 6:**
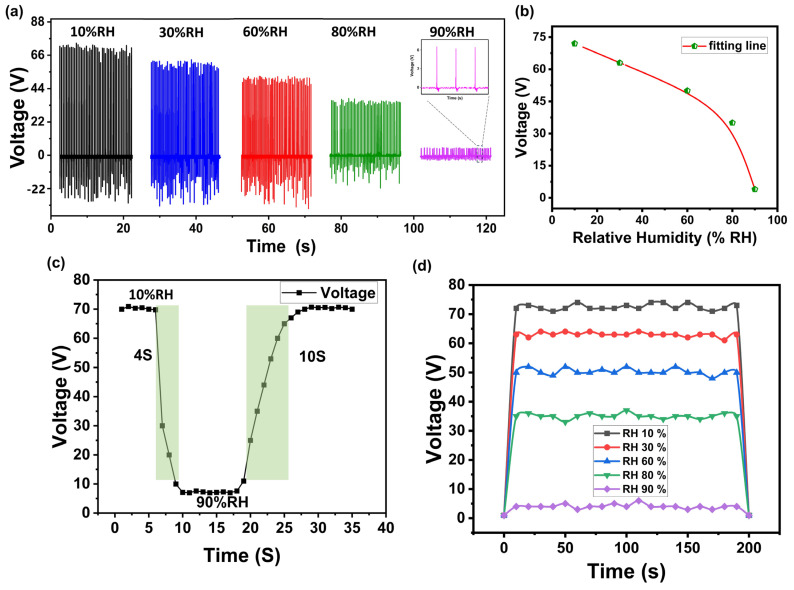
Sensor characterizations: (**a**) output voltage at RH% levels; (**b**) voltage vs. RH% fitting line; (**c**) response and recovery time; (**d**) stability test.

The humidity sensor schematic is shown in Figure 1b, where the active film of the PSP was sandwiched between the top and bottom electrodes. The resistance of this resistive humidity sensor was proportional to the environmental relative humidity (%RH). A measurement setup was constructed to investigate the humidity sensing properties of the proposed humidity sensor. The schematic diagram of the setup is shown in Figure 1c. Different standard saturated salt solutions were used to achieve the different levels of %RH. The PSP-TENG acted as the power source of the humidity sensor, and the PSP-SPHS was placed in various saturated salt solution jars to characterize it electrically at different %RH levels. The output voltage across the humidity sensor was measured by an oscilloscope. As the relative humidity (%RH) increased, there was a corresponding decrease in the resistance of the self-powered humidity sensor. This decrease in resistance led to a monotonous decrease in the output voltage across the humidity sensor, in accordance with the voltage divider principle. The relationship between %RH and resistance is such that higher humidity levels result in lower resistance, which, in turn, leads to a decrease in the output voltage of the humidity sensor. This behavior can be explained by the moisture-dependent electrical properties of the sensor material, which exhibits a sensitivity to changes in humidity levels. Figure 6a depicts the output voltage across the humidity sensor that was placed in different %RH jars. The SPHS device was successfully tested at five different %RH levels (10%, 30%, 60%, 80%, and 90% RH), and the corresponding measured output peak voltages were 70 V, 63 V, 51 V, 33 V, and 7 V, respectively. Figure 6b shows the fitting line of the average voltage across the humidity sensor at different %RH levels.

The response time of the device was tested by placing it in a 10%RH jar, and the voltage across it was constantly measured. It was then transferred to a 90%RH jar, and the time taken for the voltage to reach 90%RH was measured. Similarly, the recovery time was determined by moving the sensor from 90%RH to 10%RH. The proposed SPHS demonstrated a rapid response time of 4 s and a recovery time of 10 s. Figure 6c illustrates the response and recovery time graphs of the SPHS. Stability is a crucial characteristic of humidity sensors, and thus the stability of the fabricated device was assessed at different %RH levels. Figure 6d displays the stability test results, indicating that the SPHS maintained a consistent output voltage over an extended period at all %RH levels. The SPHS exhibited favorable performance in terms of response and recovery times while demonstrating excellent long-term stability. Table 1 summarizes the humidity sensing performance of this demonstrated PSP-SPHS with previously reported works. The response time, recovery time, sensitivity, and humidity range of PSP-SPHS were compared with those of previously reported SPHS devices made of tin disulfide nanoflowers/reduced graphene oxide [15] chitosan and activated carbon [27], graphene oxide [48], and Nb_2_CT_x_/sodium alginate [49]. The comparison showed that the PSP-SPHS had a fast response/recovery time with a greater sensitivity over a wide humidity range.

## 4. Conclusions

In this study, a bio-waste peanut skin powder (PSP) was utilized as the active layer for two distinct devices: a resistive humidity sensor and a triboelectric nanogenerator (TENG). These devices were effectively integrated to showcase a self-powered humidity sensor (SPHS). PSP was characterized by field emission scanning electron microscopy (FESEM) and Fourier transform infrared spectroscopy (FTIR) to deeply understand its surface and material characteristics. A contact separation mode TENG was fabricated using PSP and PTFE thin films as a triboelectric pair. Aluminum (Al) and copper (Cu) tape served as electrodes, and the PET sheet acted as a supporting layer on both sides of the TENG. The PSP-TENG generated an open circuit voltage of 162 V, a short circuit current of 0.2 µA, and a power of 2.2 mW, respectively, at a loading resistance of 20 MΩ. The output voltage of PSP-TENG was measured at various loading resistances and was found to increase proportionally with increasing load resistance. The rectified output of the PSP-TENG was able to charge different capacitors (10 µF to 8 nF) and powered 24 red LEDs. The fabricated PSP-based humidity sensor was resistive in nature and had a sandwich structure. The PSP thin film was sandwiched between Al and Cu electrodes. This proposed PSP-based humidity sensor was a humidity-dependent resistor whose resistance decreased with increasing %RH. The output voltage of the PSP-TENG changed as the resistance of the PSP resistive humidity sensor varied in response to the changes in humidity, utilizing the PSP-TENG as a power source. We successfully demonstrated a humidity-dependent output voltage for the proposed PSP-SPHS. The SPHS exhibited a good sensitivity of 0.8 V/%RH, along with rapid response and recovery times of 4 s and 10 s, respectively. This device also demonstrated excellent stability and repeatability, making it as a promising candidate for SPHS technology.

## Figures and Tables

**Table 1:** Comparison of PIS-SPHS with previously reported self-powered humidity sensors.

Material	Type	Response Time	Recovery Time	Sensitivity	Humidity Range	Refs.
Peanut skin powder	Triboelectric	4	10	0.8 V/RH	10~90 RH%	This work
SnS2/RGO	Triboelectric	6	15	0.25 V/RH	0~97 RH%	[15]
Graphene oxide	Piezoresistive	19	10	79.3 µV/RH	10~98 RH%	[48]
Chitosan and activated carbon	Triboelectric	12	20	Not specified	0~97 RH%	[27]
Nb_2_CT_x_/sodium alginate	Triboelectric	27	20.6	7.8 mV/RH	0~91.5	[49]

## Data Availability

Data are contained within the article and Supplementary Materials.

## Supplementary Materials

The following supporting information can be downloaded at: https://www.mdpi.com/article/10.3390/polym16060790/s1.
